# First aid in dental trauma in pediatric age

**DOI:** 10.1186/1824-7288-40-S1-A72

**Published:** 2014-08-11

**Authors:** Stefania Zampogna, Salvatore De Filippo, Valentina Talarico, Monica Aloe, Nadia Severini, Sivia Pizzi, Maria De Filippo, Antonella Polimeni

**Affiliations:** 1 Azienda Ospedaliera “Pugliese-Ciaccio”, Catanzaro, Italy; 2 Università “Magna Graecia” di Catanzaro, Catanzaro, Italy; 3 Università degli Studi di Parma, Parma, Italy; 4 Università degli Studi Roma La Sapienza, Rome, Italy

## 

The knowledge of the right management of dental traumas is very important for the pediatrician due both to their high incidence in this age group and to prevent further pathologic events related.

Several studies indicate that in industrialized countries, about one in five children have had a traumatic dental injury to permanent teeth before leaving school. Prevalence of injured teeth presented in the literature varies from 10 to 51% [1]. The pediatrician, more than any other health professionals, should have the necessary knowledge to ensure correct and professional advices for all issues concerning the child's health [2]. Most of the available literature emphasizes that awareness of the correct procedure following dental trauma is unsatisfactory. It is recognized that the prognosis of traumatic dental injuries is dependent on the time between the injury and the initiation of treatment.

**Figure 1:**
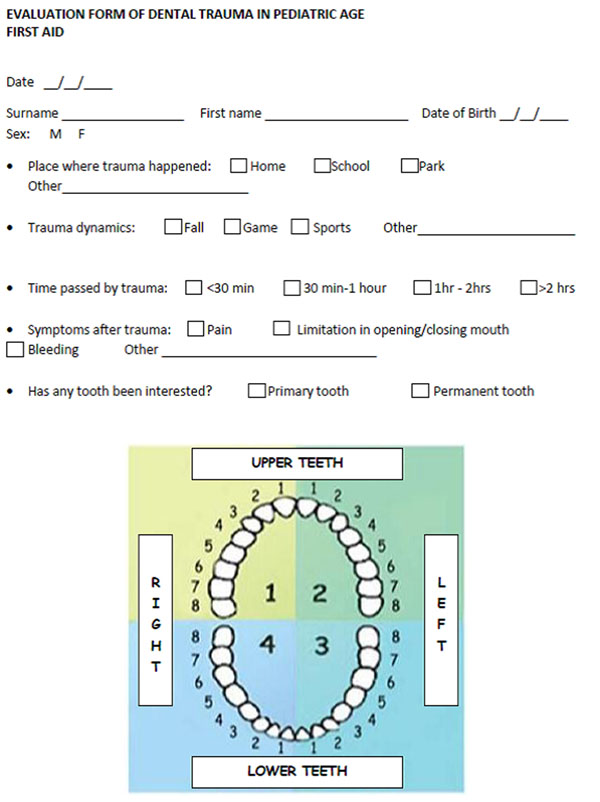
Evaluation form of dental trauma

Emergency dental treatment by a physician is sometimes required when a dentist is unavailable, so the first physician that comes to managing dental trauma is often the emergency room doctor or the family or hospital pediatrician. Some studies’ findings suggest, however, that only 4% of physicians would provide an appropriate initial treatment that could help to save an avulsed tooth [1]. Even in medical courses and first-aid training, management of dental trauma is seldom covered. To ensure and facilitate the approach to this problem, we proposed an evaluation form of dental trauma (figure 1) in order to clearly identify the points that need more attention in presence of a dental trauma and the next steps to be performed for a complete and correct clinical management [3]. First step:initial evaluation of the child, subjective information (interview, where, when, how), evaluation of vital parameters and following attribution of a color code for the priority of access to the medical examination. Next steps are the medical examination, the identification of any cranial-cervical trauma and/or signs of abuse and finally the pulpal tests.

We have proposed a multicenter study in order to evaluate the adhesion and the application of the National Guidelines for the Prevention and Clinical Management of Dental Trauma in children, published in November 2012 by the Ministry of Health. Our goal is to verify and analyze the level of knowledge of various professionals (doctors of emergency room, family pediatricians, hospital pediatricians and dentists) about the rules to prevention of dental trauma and health education, the first aid of dental trauma and recognition of dental trauma in the child abuse.
